# Induction of apoptosis in prostate carcinoma cells by BH3 peptides which inhibit Bak/Bcl-2 interactions

**DOI:** 10.1054/bjoc.2001.1850

**Published:** 2001-07

**Authors:** N M Finnegan, J F Curtin, G Prevost, B Morgan, T G Cotter

**Affiliations:** Dept of Biochemistry, University College Cork, Lee Maltings, Prospect Row, Cork, Ireland; Beaufour Ipsen, Institut Henri Beaufour, 5 Avenue du Canada, Les Ulis Cedex, 91966, France; Biomeasure Inc., 27 Maple St., Milford, MA

**Keywords:** prostate, apoptosis, BH3, Bcl-2, peptide

## Abstract

Interactions between proteins of the Bcl-2 family play an important role in the regulation of apoptosis. Anti-apoptotic family members can heterodimerize with pro-apoptotic family members and antagonize their function, thus protecting against death. In cells protected from death by overexpression of Bcl-2 much of the Bax is present in Bax/Bcl-2 hetero-multimers and its death signal is blocked as it cannot homodimerize. This led us to use the Bcl-2/Bax heterodimer as a target for new compounds which may provide a therapy particularly suited to tumour cells for which resistance to conventional therapy is associated with elevated expression of Bcl-2. We assessed whether apoptosis could be induced in prostate tumour cells by blocking this heterodimerization with synthetic peptide sequences derived from the BH3 domain of pro-apoptotic Bcl-2 family members. Prostate cells were found to undergo up to 40% apoptosis 48 h following the introduction of synthetic peptides from the BH3 domains of Bax and Bak. The caspase inhibitor z-VAD.fmk provided protection against apoptosis mediated by these peptides. Immunoprecipitation studies revealed that introduction of peptides derived from the BH3 regions of Bak and Bax into cells blocked Bak/Bcl-2 heterodimerization. These data suggest that by blocking the dimerization through which Bcl-2 would normally inhibit apoptosis the apoptotic pathway driven by Bak was re-opened. © 2001 Cancer Research Campaign http://www.bjcancer.com


British Journal of Cancer (2001) **85**(1), 115–121
© 2001 Cancer Research Campaign
doi: 10.1054/ bjoc.2001.1850, available online at http://www.idealibrary.com on IDEAL®

http://www.bjcancer.com

### Induction of apoptosis in prostate carcinoma cells by BH3 peptides which inhibit Bak/Bcl-2 interactions

**NM Finnegan[1], JF Curtin[1], G Prevost[2], B Morgan[3] and TG Cotter[1]**

[1]Dept of Biochemistry, University College Cork, Lee Maltings, Prospect Row, Cork, Ireland; [2]Beaufour Ipsen, Institut Henri Beaufour, 5 Avenue du Canada, 91966, Les Ulis Cedex, France; [3]Biomeasure Inc., 27 Maple St., Milford, MA

**Summary** Interactions between proteins of the Bcl-2 family play an important role in the regulation of apoptosis. Anti-apoptotic family members can heterodimerize with pro-apoptotic family members and antagonize their function, thus protecting against death. In cells protected from death by overexpression of Bcl-2 much of the Bax is present in Bax/Bcl-2 hetero-multimers and its death signal is blocked as it cannot homodimerize. This led us to use the Bcl-2/Bax heterodimer as a target for new compounds which may provide a therapy particularly suited to tumour cells for which resistance to conventional therapy is associated with elevated expression of Bcl-2. We assessed whether apoptosis could be induced in prostate tumour cells by blocking this heterodimerization with synthetic peptide sequences derived from the BH3 domain of pro-apoptotic Bcl-2 family members. Prostate cells were found to undergo up to 40% apoptosis 48 h following the introduction of synthetic peptides from the BH3 domains of Bax and Bak. The caspase inhibitor z-VAD.fmk provided protection against apoptosis mediated by these peptides. Immunoprecipitation studies revealed that introduction of peptides derived from the BH3 regions of Bak and Bax into cells blocked Bak/Bcl-2 heterodimerization. These data suggest that by blocking the dimerization through which Bcl-2 would normally inhibit apoptosis the apoptotic pathway driven by Bak was re-opened. © 2001 Cancer Research Campaign http://www.bjcancer.com

**Keywords**: prostate; apoptosis; BH3; Bcl-2; peptide

Carcinoma of the prostate is the second most common cause of cancer-related deaths in males (Tang and Porter 1997). The disease occurs in 2 phases, with the first phase characterized by the growth of hormone-dependent tumours which respond to hormone ablation therapy, and in the second phase, usually 2–3 years post-therapy, the disease re-appears in the form of hormone-insensitive tumours which are unresponsive to conventional chemotherapy (Kreis, 1995). Improved understanding of the mechanism(s) by which hormone-independent prostate tumour cells become resistant to death mediated by conventional anti-cancer agents may provide the basis for the design of new therapeutic approaches to the treatment of advanced prostate cancer.

Apoptosis is defined as programmed cell death or cell suicide and is a process in which the cell actively participates in its own destruction, under the control of a variety of internal and external signals. The apoptotic cell undergoes several morphological changes in this complicated pathway to cell death including cytoplasmic dehydration and shrinkage, chromatin condensation and the formation of apoptotic bodies containing chromatin and cytoplasm in the final stages (Mundle et al, 1996). Since rates of cell proliferation relative to cell death are crucial to the homeostasis of an organism, abnormal apoptotic rates are prevalent in human diseases, and either a lack of or too much apoptotic death may lead to cancer or degenerative disease, respectively (Allen et al, 1998).

Of the proteins and genes involved in the regulation of apoptosis, members of the Bcl-2 gene family play a central role in its inhibition or promotion. In mammalian cells this polypeptide family comprises several members, some of which suppress cell death, e.g., Bcl-2 and Bcl-$x_L$ while others promote apoptosis, e.g., Bax, Bik, Bid and Bak (Kroemer, 1997). Nearly all Bcl-2 family member have a membrane-targeting domain at its carboxy terminus allowing it to attach to mitochondrial, ER and nuclear membranes (Zhu et al, 1996). Pro-and anti-apoptotic Bcl-2 family members can form homodimers and heterodimers and the relative ratios of these dimers are believed to gauge the sensitivity of a cell towards either survival or apoptosis. Bcl-2 family members share at least one of the Bcl-2 homology regions 1, 2, 3 and 4 (BH1, BH2, BH3 and BH4) (Yang and Korsmeyer, 1996) which are required for dimerization and for regulation of apoptosis (Hirotani et al, 1999). Truncated forms of pro-apoptotic Bak (Chittenden et al, 1995) and Bax (Han et al, 1996) consisting only of their BH3 regions have been shown to be sufficient to induce apoptosis and antagonize the effects of anti-apoptotic members (Zhou et al, 2000).

In the second phase of prostate cancer apoptosis-resistant clones appear as a result of genetic alterations (Tang and Porter, 1997). Bcl-2 is commonly overexpressed in a number of cancers, including hormone-independent prostate carcinoma (Colombel et al, 1993; Krajewska et al, 1996), and it has been indicated that androgens promote Bcl-2 expression (Berchem et al, 1995). Also, prostate cancer patients with an elevated Bcl-2/Bax ratio are associated with an increased risk of their cancer failing to respond to radiotherapy (Mackey et al, 1998). The ability of Bcl-2 to block apoptosis may be an important factor in the development of this disease from the hormone-dependent form to the more aggressive hormone-independent form.

We hypothesized that the introduction of synthetic BH3 peptides into cells may increase levels of apoptosis in tumour cells

*Received 27 November 2000*
*Revised 26 March 2001*
*Accepted 29 March 2001*

*Correspondence to:* TG Cotter by binding to the region on Bcl-2 corresponding to the position where pro-apoptotic Bcl-2 family members bind, thus removing the inhibitory effect on pro-apoptotic family members, leaving them free to mediate apoptosis. In an attempt to combat the apoptosis resistance associated with prostate cancer metastasis, we chose a model where synthetic peptides from the BH3 domain of pro-apoptotic Bcl-2 family members were introduced into Bcl-2-expressing, hormone-independent prostate cells. We demonstrated that this resulted in the induction of apoptosis and showed that the probable mode of action of the peptides was through their modulation of the interactions of Bcl-2 family members such that the dominant influence on cell fate switched from the anti-apoptotic to the apoptosis-inducing family members, resulting in cell death.

### MATERIALS AND METHODS

### Tumour cells and culture conditions

The human hormone-independent cell lines PC-3 and DU145 were obtained from American Type Culture Collection (Rockville, MD). Cells were grown in humidified atmosphere at 37˚C in 5% $CO_2$ and were maintained in culture as adherent cells in RPMI 1640 (Gibco Life Technologies, Paisley, Scotland) containing 10% FCS (Gibco), L-glutamine and antibiotics.

### Reagents and antibodies

Peptides (15 amino acids) derived from the BH3 domains of apoptosis agonists Bax (native and C->S forms), Bak and Bik (native and C->S forms) were provided by Biomeasure Inc, MA and stock solutions in DMSO (BDH Laboratory Supplies, Poole, UK) were prepared at 10 mg $ml^{-1}$ and stored at room temperature. FITC-conjugated annexin V was purchased from Bender MedSystems (Vienna, Austria) and propidium iodide (PI) from Sigma Chemical Co (St Louis, MO). The following antibodies were used in the immunoblotting experiments; a mouse monoclonal anti-human antibody to Bcl-2 (amino acids 41–54 as immunogen) from Dako (clone 124) (Cambridge, UK), a mouse monoclonal anti-human antibody to Bak (amino terminal portion) from Calbiochem (San Diego, CA) and a mouse monoclonal antibody to Bax (recombinant human Bax) from Immunotech (Coulter, Miami, FA). The secondary antibody used for immunoblotting was an anti-mouse IgG (whole molecule) conjugated to HRP (Dako). Benzyloxycarbonyl-valinyl-alaninyl-aspartyl-(O-methyl)-fluoromethylketone (z-VAD.fmk) was obtained from Enzyme Systems Products (Livermore, CA).

### Transfer of BH3 peptides into cells via electroporation

PC3 and DU145 cells were harvested during the exponential growth phase by trypsinization and were resuspended in their original media. They were washed in 0.015 M PBS, pH 7.2 (0.14 M NaCl, 2 mM KCl, 8 mM $Na_2HPO_4$ and 1.5 mM $NaH_2PO_4$) and resuspended at a concentration of $0.625 \times 10^6$ cells $ml^{-1}$ in PBS. PBS was found to give optimum results as an electroporation buffer as compared with HEPES (Gibco) buffer (20 mM) or RPMI 1640, and was used throughout as the standard electroporation buffer. 0.8 ml of the cell suspension was mixed with the peptides, allowed to stand at room temperature for 10 min and added to a disposable 0.4 cm Bio-Rad electroporation cuvette. An equivalent volume of DMSO was added to a cell suspension without peptide for use as a control. Electroporation efficiency for each cell line was initially determined flow cytometrically by uptake of the fluorescent dye, lucifer yellow (Sigma). Electroporation was carried out in a Gene-Pulser (Bio-Rad) with cells exposed to one pulse. Following electroporation, cells were allowed to recover by standing at room temperature for 10 min, then removed from the electroporation chamber, washed twice in PBS and resuspended in 2 ml of complete RPMI 1640 at 37˚C prior to analysis.

### Detection of apoptosis by FITC-labelled Annexin V binding

PC-3 and DU145 cells were incubated for up to 48 hours with BH3 peptides under the usual cell culture conditions. Apoptosis levels were assessed flow cytometrically in a Becton Dickinson FACScan by PI uptake and annexin V binding. Annexin V binds to phosphatidylserine present on the outer leaflet of the plasma membrane in early apoptotic cells. Viable cells were identified by their ability to exclude PI. Control and treated cells were washed once with PBS by centrifugation at 400 ***g***, resuspended in HEPES buffer (10 mM HEPES-NaOH, pH 7.4, 150 mM NaCl, 5 mM KCl, 12 mM $MgCl_2$ and 1.8 mM $CaCl_2$), incubated with FITC-conjugated annexin V (1 μg $ml^{-1}$) (Bender MedSystems) and PI (10 μg $ml^{-1}$) (Sigma) for 5 min at room temperature and immediately analysed by flow cytometry to quantitate annexin V binding (FL-1) and PI uptake (FL-2). A minimum of 10 000 events were analysed for each sample.

### Immunoprecipitation and Western blotting

PC-3 cells were lysed in 0.2% NP-40 isotonic lysis buffer as previously described (Oltvai et al, 1993). Immunoprecipitation was carried out with 300 μg of cell lysate. For immunoprecipitation of Bcl-2, a mouse monoclonal anti-human Bcl-2 antibody (specific for Bcl-2 p25) coupled to agarose (Santa Cruz Biotechnology) was added to each sample. For immunoprecipitation with Bak and Bax, lysates were precleared with goat anti-mouse IgG agarose (Sigma) which was removed by centrifugation. Specific antibodies (anti-Bak or anti-Bax) and goat anti-mouse IgG agarose were used to capture the immune complexes. Western blot analysis was subsequently carried out.

### RESULTS

### Western blot analysis for the expression of Bcl-2 family members in the PC-3 prostatic carcinoma cell line

A therapy strategy based on interfering with the molecular interactions of Bcl-2 and its proapoptotic counterparts would require that the target cells used express the appropriate members of the Bcl-2 family of proteins. Previous studies have shown that PC-3 cells are positive for several Bcl-2 family member proteins (Haldar et al, 1996; Rokhlin et al, 1997). Cells with high levels of expression of anti-apoptotic family members, particularly Bcl-2 or Bcl-$x_L$, could be expected to respond optimally to this mode of therapy.

We examined by Western blot analysis the presence of Bcl-2 family members in the PC-3 and DU145 cell lines. Analysis revealed the presence of Bcl-2, Bcl-$X_L$, Bax and Bak in PC-3 cells. We found that DU145 cells expressed low levels of Bcl-$X_L$ and Bax, but expressed high levels of Bcl-2 and Bak (Figure 1).

 © 2001 Cancer Research Campaign

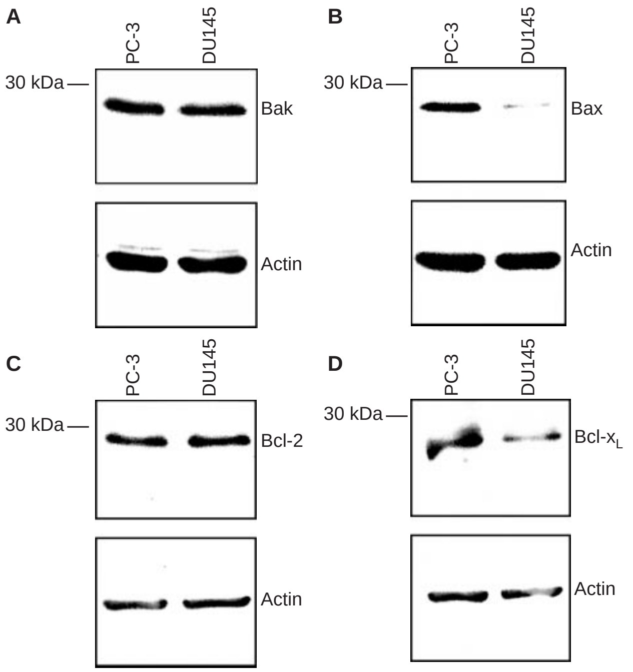

**Figure 1** Expression of various Bcl-2 family members in lysates from PC-3 and DU145 cells. Cellular protein lysates were resolved by 12% SDS-PAGE under reducing conditions, transferred to nitrocellulose membranes and detected by the corresponding Abs. Bak (**A**), Bax (**B**), Bcl-2 (**C**) and Bcl-$X_L$ (**D**) were probed on 4 separate blots as the molecular weights of the Bcl-2 family members were similar. Actin was also probed to ensure equal loading

### Introduction of BH3 peptides into prostatic tumour cells by electroporation induced apoptosis

Electroporation conditions for PC-3 and DU145 cells were initially optimized for efficiency of delivery of macromolecules into the cell by the use of the fluorescent marker lucifer yellow. Conditions were set at 25 microfarads and 0.6 kV $cm^{-1}$ for PC-3 cells and at 960 microfarads and 0.15 kV $cm^{-1}$ for DU145 cells which gave extensive intracellular delivery at the lowest expense of viability (≤80%), as assessed by PI uptake. At higher voltages extensive cell lysis was observed.

These settings were then used to evaluate the effect of electroporation of peptides derived from the BH3 domains of pro-apoptotic Bcl-2 family members (Table 1) on apoptosis levels of PC3 and DU145 cells as compared to control cells electroporated under identical conditions in the absence of peptide. Electroporation of PC-3 cells with peptides mimicking the BH3 regions of Bak and Bax (C->S form) resulted in the induction of apoptosis over time (Figure 2A, B), at a peptide concentration of 50 μg $ml^{-1}$. At 10 h cells exhibited apoptotic features which were detected by changes in PS on the plasma membrane as measured by annexin V uptake, reaching 40–50% apoptosis 48 h after introduction of the peptides. Under identical electroporation conditions peptides from the BH3 regions of Bik (native and C->S form) and of Bax showed no comparable increase in apoptosis in PC-3 cells (Figure 2C). Apoptosis levels were the same as control levels in cells exposed to BH3 peptides in the absence of electroporation (Figure 2D), indicating that intracellular delivery of BH3 peptides was required for demonstration of the apoptotic effect of BH3 peptides. Compared to control cells electroporated under identical conditions in the absence of peptide. Expression of Bcl-2 family

**Table 1** Peptides derived from BH3 domains

| BH3 domain | Sequence[a] | | |
|---|---|---|---|
| Bak | Ac 74 VGRQLAIIGDDINRR | 88 | NH2 |
| Bax | Ac 59 LSECLKRIGDELDSN | 73 | NH2 |
| Bik | Ac 57 LALRLACIGDEMDVS | 71 | NH2 |
| Bax (C62->S)[b] | Ac 59 LSESLKRIGDELDSN | 73 | NH2 |
| Bik (C63->S)[b] | Ac 57 LALRLASIGDEMDVS | 71 | NH2 |

[a]Sequences are shown using the single letter code for amino acids. Numbers refer to the position of the terminal amino acids of the peptide in the parent protein. [b]We found that native Bax and Bik peptides did not induce any apoptosis in the cells. We discovered that the cysteine residues in the sequences were unstable and were oxidised, producing unactive peptides. By creating the Bax(C62->S) and Bik (C63->S) peptides we found that the peptides were more stable in storage and the Bax (C62->S) peptide was capable of inducing apoptosis in both PC-3 cells and DU145 cells.

members has been previously examined by Western Blot analysis in the DU145 prostate cell line (Rokhlin et al, 1997) and it has been shown to express Bcl-$X_L$ and Bak. We found that although DU145 cells express both Bak and Bcl-$X_L$, Bcl-$X_L$ is only expressed in low amounts and it appears that Bcl-2 is the more important anti-apoptotic family member in these cells (Figure 1). The effects of electroporation of DU145 cells with the BH3 peptides (derived from Bak and Bax) was also assessed over time (Figure 2E, F), and results showed that up to 40% apoptosis was induced after 48 h at a peptide concentration of 50 μg $ml^{-1}$.

### The caspase inhibitor z-VAD.fmk reduced apoptosis induced by BH3 peptides to control levels

While we had already shown that tumour cells underwent changes in PS on the plasma membrane in response to electroporation of BH3 peptides, a typical feature of an apoptotic cell, we wished to further confirm that the effects seen were as a result of apoptosis. Caspase activation is a key event in the process of apoptosis and we next assessed whether, by inhibiting caspase activity in PC-3 and DU145 cells, apoptosis was modulated. In order to demonstrate whether caspases played a role in the apoptotic pathway mediated by BH3 peptides a broad spectrum peptide inhibitor of the caspase family, z-VAD.fmk (50 μM), was added to cells just prior to electroporation with the peptides and apoptosis levels over time were measured as before by surface expression of FITC-labelled annexin V. Cells that were treated with peptides in the presence of z-VAD.fmk had the inhibitor present in the culture medium throughout the duration of the incubation period.

As shown in Table 2, addition of z-VAD.fmk to PC-3 and DU145 cells afforded protection from apoptosis and led to the abrogation of the apoptotic effects of peptides from the BH3 regions of both Bax and Bak, leaving apoptosis levels equal to control cells electroporated in the absence of peptide. This result indicated that apoptosis induced in cells following the introduction of BH3 peptides by electroporation acted via activation of a z-VAD.fmk-sensitive agent, presumably one or more members of the caspase family.

Also of note in Table 2 is that the peptide from the BH3 region of Bax which did not induce apoptosis following electroporation showed was no change in this level of apoptosis upon treatment with z-VAD.fmk. This result indicated that the inhibitory effect of z-VAD.fmk is specific for apoptosis induced by peptides from the BH3 regions of Bak and Bax (C->S form).

*© 2001 Cancer Research Campaign* 

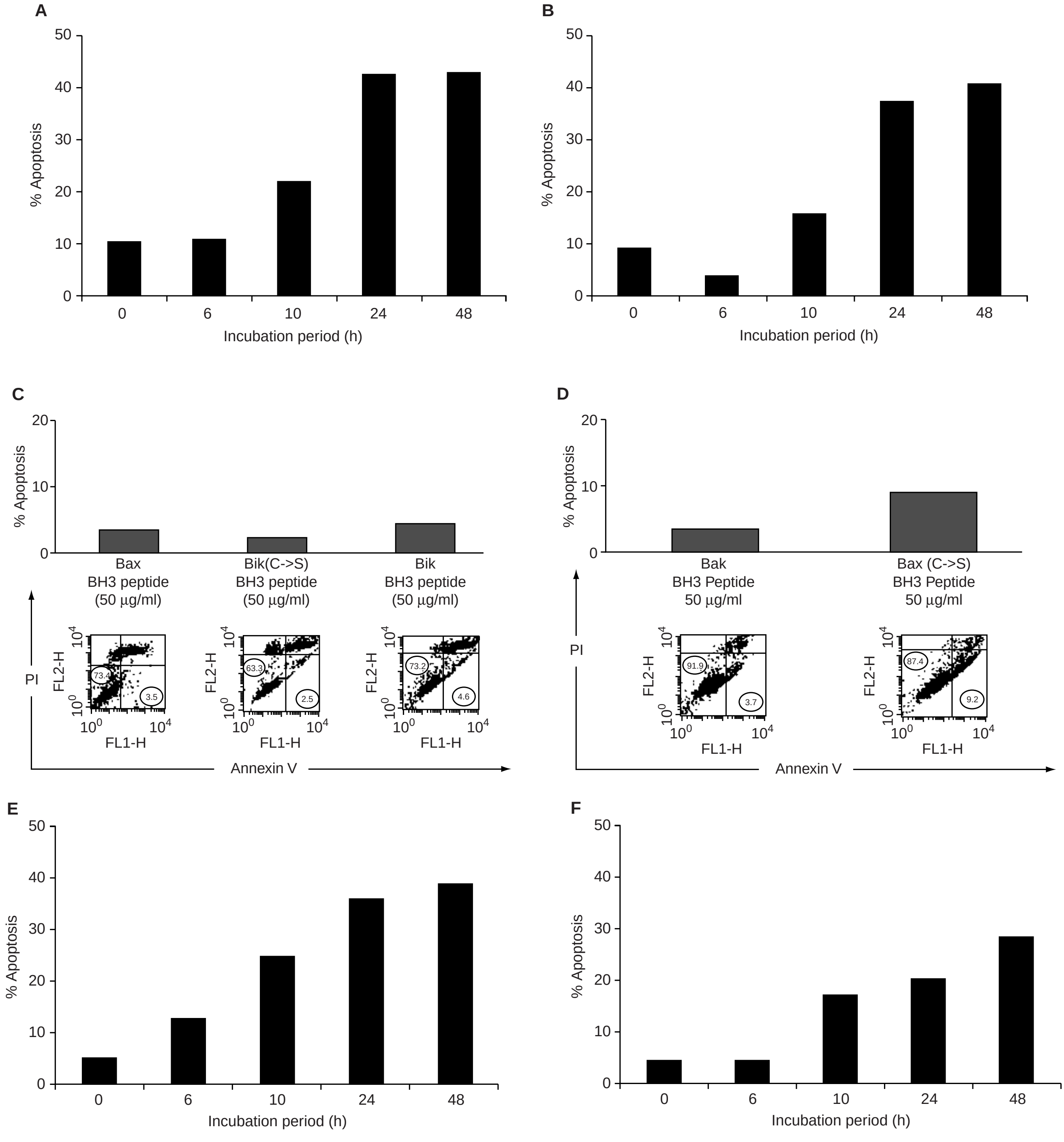

**Figure 2** The effects of BH3 peptides on apoptosis levels in prostate cancer cell lines. Analysis of the levels of apoptosis over time of PC-3 cells after electroporation with peptides derived from (**A**) the BH3 domain of Bak (50 µg ml$^{-1}$) and (**B**) the BH3 domain of Bax(C->S) (50 µg ml$^{-1}$), (**C**) the BH3 domains of Bax and Bik (native and C->S forms) (50 µg ml$^{-1}$) at T = 18 h, (**D**) shows the levels of apoptosis at T = 24 h in cells exposed to BH3 peptides in the absence of electroporation and finally the levels of apoptosis over time of DU145 cells after electroporation with peptides derived from the BH3 domains of (**E**) Bak (50 µg ml$^{-1}$) and (**F**) Bax (C->S) (50 µg ml$^{-1}$). Cells ($0.5 \times 10^6$) were electroporated with or without BH3 peptides and apoptosis was assessed immediately (T = 0 h) or cells were then incubated in RPMI1640 at 37˚C and harvested 6 h, 10 h, 24 h and 48 h after electroporation. The data are representative of at least 3 separate experiments. Apoptosis was determined by staining cells with FITC-labelled annexin V (FL1) and PI (FL2). Apoptosis was assayed by FACS analysis and the percentage of cells undergoing apoptosis (represented in the lower right hand quadrants in **C** and **D**) were measured for up to 48 h

### Immunoprecipitation studies showed no evidence of heterodimerization between Bcl-2 and Bax

Pro- and anti-apoptotic Bcl-2 family members can heterodimerize and seemingly titrate one another's function (Adams and Cory, 1998). Bcl-2 and Bax have been shown to heterodimerize in several cell lines (Yin et al, 1994; Otter et al, 1998) and both proteins were found to be present in PC-3 cells. Following the finding that a peptide derived from the BH3 region of Bax mediated apoptosis in PC-3 cells we next assessed whether this was

 *© 2001 Cancer Research Campaign*

**Table 2** Effects of Z-VAD.fmk on levels of apoptosis in PC-3 and DU145 cells following electroporation with BH3 peptides

| | **PC-3** | **DU145** |
|---|---|---|
| **BH3 domain** | **% Apoptosis**[a] | |
| Control | 10.1 ± 1.00 | 6.4 ± 3.74 |
| Bax(C62->S) BH3 domain (50 µg ml$^{-1}$) | 19.2 ± 1.80 | 20.2 ± 1.08 |
| Bax(C62->S) BH3 domain + Z-VAD | 9.3 ± 0.40 | 8.4 ± 1.44 |
| Bak BH3 domain (50 µg ml$^{-1}$) | 23.1 ± 5.03 | 25.5 ± 4.16 |
| Bak BH3 domain + Z-VAD | 12.9 ± 0.60 | 11.2 ± 1.69 |
| Bax BH3 domain (50 µg ml$^{-1}$) | 11.1 ± 0.85 | 11.6 ± 2.60 |
| Bax BH3 domain + Z-VAD | 11.1 ± 0.55 | 6.4 ± 0.53 |

[a]Cells were electroporated with peptides from the BH3 regions of Bax and Bak, incubated with or without ZVAD.fmk (50 µM) for 10 h and apoptosis was measured flow cytometrically by assessment of uptake of FITC-labelled annexin V and PI. Results show the mean of 3 separate experiments ± SD.

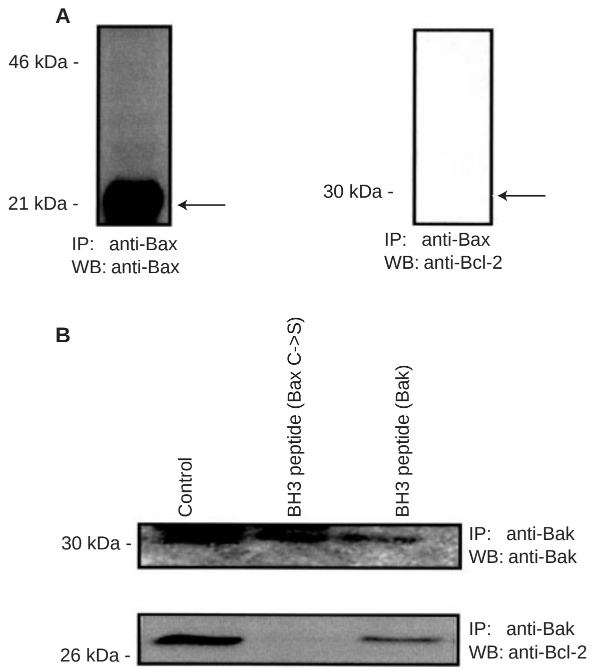

**Figure 3** Immunoprecipitation of PC-3 cells to look for Bax/Bcl-2 and Bak/Bcl-2 heterodimerization. (**A**) Cells were immunoprecipitated with anti-Bax mAb, divided into equal portions and loaded onto duplicate SDS-PAGE gels and immunoblotted with anti-Bcl-2 and anti-Bax antibodies as detailed in the Materials and Methods section. (**B**) Immunoprecipitation of PC-3 cells following electroporation of BH3 peptides. Cells were electroporated with BH3 peptides (50 µg ml$^{-1}$), whole cell lysates were extracted and 300 µg of lysates were immunoprecipitated with anti-Bak mAb. The reconstituted immune complexes were divided equally and loaded onto duplicate 10% SDS-PAGE gels and immunoblotted with either anti-Bak or anti-Bcl-2 mAb associated with altered interactions between Bcl-2 and Bax in these cells. We first determined whether heterodimerization between Bcl-2 and Bax could be detected in the PC-3 cell line by immunoprecipitation studies. Results showed that, while PC-3 cells immunoprecipitated with an anti-Bax antibody yielded large amounts of Bax protein, probing this immunoprecipitated Bax with an anti-Bcl-2 antibody showed that the 2 proteins did not appear to co-immunoprecipitate (Figure 3a). Based on this and subsequent experimental observations we concluded that there was no detectable heterodimerization of Bcl-2 and Bax proteins in PC-3 cells.

### Immunoprecipitation studies showed heterodimerization between Bcl-2 and Bak and evidence of modulation of this interaction by BH3 peptides

Since the peptide mimicking the BH3 region of Bak was found to induce apoptosis in PC-3 cells we next assessed by co-immunoprecipitation studies whether there was an association between Bcl-2 and Bak in unstimulated, control cells and whether the introduction by electroporation of BH3 peptides into the cytoplasm of PC-3 cells antagonised the association between Bcl-2 and Bak.

Immunoprecipitation studies were carried out on lysates from untreated PC-3 cells and cells electroporated with peptides derived from the BH3 regions of Bak and Bax. Figure 3B demonstrates that the level of Bak immunoprecipitated from untreated cells and cells electroporated with BH3 peptides are comparable. However, when the same samples were probed to assess whether Bcl-2 co-immunoprecipitated with Bak, it was found that the levels of Bcl-2 which were associated with Bak varied greatly between the 3 groups. Untreated cells showed a strong association between Bak and Bcl-2, indicating that these proteins heterodimerize in the control state. Levels of Bcl-2 found to co-immunoprecipitate with Bak were greatly reduced in PC-3 cells electroporated with peptide from the BH3 region of Bak compared with control cells, while treatment with peptide from the BH3 region of Bax appeared to abrogate all Bak/Bcl-2 interactions. This result demonstrated that the introduction via electroporation of a peptide derived from the BH3 region of Bak into PC-3 cells strongly inhibited interactions between Bak and Bcl-2 compared with control cells and that this interaction was completely abrogated following the introduction of a peptide derived from the BH3 region of Bax.

### DISCUSSION

The purpose of this study was to determine whether apoptosis could be induced in an androgen-independent prostate carcinoma cell line by introducing synthetic peptides derived from the BH3 regions of pro-apoptotic Bcl-2 family members into the cells. Prostate cancer progression and metastasis is associated with over-expression of Bcl-2 and the acquisition of an androgen-insensitive phenotype that is resistant to current therapies (McDonnell et al, 1992), suggesting that apoptosis resistance may be a cause of drug resistance.

Here we have shown that apoptosis was induced in prostate carcinoma cell lines by introducing peptides from the BH3 regions of Bax and Bak and that, when Bak was immunoprecipitated from lysates of cells treated with a peptide mimicking the BH3 region of Bak, a significant reduction in the amount of Bcl-2 co-immunoprecipitating with Bak was detected. This indicated the likelihood that, in this system, Bcl-2/Bak heterodimers were required for the potent death suppressor activity of Bcl-2 and that by introducing BH3 peptides into prostate tumour targets this suppression could be attenuated. The resulting increase in levels of apoptosis could be due to the release of Bax or Bak-like proteins from the inhibitory influence of Bcl-2-like proteins, resulting in cell death.

*© 2001 Cancer Research Campaign* 

The BH3 regions of Bak and Bax are required for promoting cell death and for binding to apoptotic inhibitors (Chittenden et al, 1995; Zha et al, 1996), however, evidence suggests that apoptosis antagonists of the Bcl-2 family protect cells from death by binding to and neutralizing death agonists (Yin et al, 1994; Allen et al, 1998). Mutations within the BH domains of Bcl-2 and Bcl-$x_L$ can disrupt heterodimerization with proapoptotic family members (Yin et al, 1994) and it has previously been shown that a 50% reduction in the formation of Bcl-2/Bax heterodimers can drive cells towards apoptosis (Yang et al, 1995; Haldar et al, 1996). Thus, our hypothesis was that peptides corresponding to the BH3 region of pro-apoptotic Bcl-2 family members could interfere with protein–protein interactions between Bcl-2 family members such that the dominant influence on cell fate was switched from the anti-apoptotic to the apoptosis-inducing family members.

We have shown that treatment of cells with peptides mimicking the BH3 region of Bax resulted in the abrogation of Bak/Bcl-2 interactions. Bak and Bax share significant sequence homology (Diaz et al, 1997; Kroemer, 1997) and they share common targets, e.g., the Bcl-2 protein. While it is possible that the peptides bind preferentially to the proapoptotic Bcl-2 family members we feel that this is unlikely in the context of our data. Our results indicated that peptides from the BH3 regions of both Bax and Bak bound with Bcl-2, displacing Bcl-2/Bak heterodimer formation, and implied that the peptide from the BH3 region of Bax had a higher affinity for heterodimerization with Bcl-2 than the peptide from the BH3 domain of Bak. Pro-apoptotic Bad has been shown to oppose Bcl-$x_L$-mediated survival and accelerate cell death by displacing Bax from Bcl-$x_L$ (Yang et al, 1995). In a similar fashion, BH3 peptides may have a high affinity for heterodimerization with Bcl-2 and consequently displace Bak/Bcl-2 heterodimers. Peptides derived from the BH3 domains of Bak and Bax block both Bcl-2/Bax and Bcl-2/Bcl-2 binding (Diaz et al, 1997). It thus appears that many of the Bcl-2 family members share common binding motifs that are responsible for homodimerization and heterodimerization and it is likely that the BH3 peptides can modulate interactions involving other anti-apoptotic family members, e.g., Bcl-$x_L$, and contribute to the apoptotic effect seen here.

It has previously been shown that pro-apoptotic Bak and Bik play a role in the initiation of the apoptotic programme and that z-VAD.fmk attenuated this cell death, implying that these Bcl-2 family members trigger apoptosis by activation of caspases (Orth et al, 1997). When we investigated the ability of the broad specificity caspase inhibitor, z-VAD.fmk, to inhibit apoptosis triggered by BH3 peptides in PC-3 cells we found that, in the case of peptides resembling the BH3 regions of Bak and Bax, z-VAD.fmk provided complete protection from apoptosis. Thus, if the apoptosis induced by BH3 peptides is mediated by the pro-apoptotic members of the Bcl-2 family such as Bak, then this result implies that they mediate apoptosis through activation of a z-VAD.fmk-sensitive regulator, which indicates a downstream effector role for caspases. Pan et al have shown that Bcl-$X_L$ and not Bcl-2 sequesters APAF-1, a vital component of the Caspase 9 apoptosome (Pan et al, 1998). Thus the Bcl-2 family members inhibit apoptosis through a variety of mechanisms. Further study would be required to delineate the apoptotic pathway following incubation of Prostate cancer cells with BH3 peptides. The actual caspases involved remains to be elucidated but the mechanism is most likely mediated through caspase 9 activation and subsequent caspase 3 activation (Jurgensmeier et al, 1998). Similar to reports by other authors who found that Bax-mediated release of cytochrome *c* was not accompanied by mitochondrial permeability transition (Jurgensmeier et al, 1998), we did not see changes in mitochondrial depolarization following introduction of BH3 peptides (data not shown).

Our results are in agreement with the hypothesis that BH3 peptides interact with the site on Bcl-2 where pro-apoptotic proteins bind, thus preventing the subsequent binding of BH3 domains of Bak or Bax-like proteins to these sites and resulting in the liberation of Bak/Bax to mediate cell death as homodimers/monomers. In conclusion, we have shown that peptides mimicking the BH3 domains of pro-apoptotic Bcl-2 family members Bak and Bax-induced apoptosis in prostate carcinoma cell lines via a z-VAD.fmk-inhibitable mechanism. While the BH3 peptides themselves are not cell permenant, we are currently developing an assay to identify non-peptidic compounds which mimic the properties of these peptides. These results point to the potential use of such agents as a new therapeutic strategy for the treatment of diseases associated with resistance to apoptosis. It would be necessary to establish whether the anti-apoptotic effects of these compounds are restricted to prostate tumour cells by examining their efficacy in the treatment of other human neoplasms. BH3 peptide therapy may have a distinct advantage over current treatment regimens in that cells in which apoptosis is suppressed by elevated levels of anti-apoptotic Bcl-2 family members are selectively killed. However, in order to establish the clinical relevance of these compounds, their efficacy would need to be compared with the efficacy of other clinically relevant treatment regimens that induce apoptosis in their tumour targets, such as chemotherapeutic agents and radiation.

### ACKNOWLEDGEMENTS

This study was supported by Biomeasure Inc.

### REFERENCES

Adams JM and Cory S (1998) The Bcl-2 protein family: Arbiters of cell survival. *Science* **281**: 1322–1326

Allen RT, Cluck MW and Agrawal DK (1998) Mechanisms controlling cellular suicide: role of Bcl-2 and caspases. *Cell Mol Life Sci* **54**: 427–445

Berchem GJ, Bosseler M, Sugars LY, Voeller HJ, Zeitlin S and Gellmann EP (1995) Androgens induce resistance to Bcl-2-mediated apoptosis in LNCaP prostate cancer cells. *Cancer Res* **55**: 735–738

Chittenden T, Flemmington C, Houghton AB, Ebb RG, Gallo GL, Elangovan B, Chinnadurai G and Lutz RJ (1995) A conserved domain in Bak, distinct from BH1 and BH2, mediates cell death and protein binding function. *EMBO J* **14**: 5589–5596

Colombel M, Symmans F, Gil S, O'Toole KM, Chopin D, Benson M, Olsson CA, Korsmeyer S and Buttyan R (1993) Detection of the apoptosis-suppressing oncoprotein bcl-2 in hormone refractory human prostate cancers. *Am J Pathol* **143**: 390–400

Diaz JL, Oltersdorf T, Horne W, McConnell M, Wilson G, Weeks S, Garcia T and Fritz CF (1997) A common binding site mediates heterodimerization and homodimerization of Bcl-2 family members. *J Biol Chem* **272(17)**: 11350–11355

Haldar S, Chintapalli J and Croce CM (1996) Taxol induces *Bcl-2* phosphorylation and death in prostate cancer cells. *Cancer Res* **56**: 1253–1255

Han JP, Sabbatini P, Perez D, Rao L, Modha D and White E (1996) The E1B 19K protein blocks apoptosis by interacting with and inhibiting the p53-inducible and death-promoting Bax protein. *Genes Dev* **10**: 461–477

Hirotani M, Zhang Y, Fujita N, Naito M and Tsuruo T (1999) NH2-terminal BH4 domain of Bcl-2 is functional for heterodimerization with Bax and inhibition of apoptosis. *J Biol Chem* **274**: 20415–20420

Jurgensmeier JM, Xie Z, Deveraux Q, Ellerby L, Bredesen D and Reed JC (1998) Bax directly induces release of cytochrome *c* from isolated mitochondria. *Proc Natl Acad Sci USA* **95**: 4997–5002

 *© 2001 Cancer Research Campaign*

Krajewska M, Krajewski S, Epstein JI, Shabaik A, Sauvageot J, Song K, Kitada S and Reed JC (1996) Immunohistochemical analysis of bcl-2, bax, bcl-$x_L$, and mcl-1 expression on prostate cancers. *Am J Pathol* **148**: 1567–1576

Kreis W (1995) Current chemotherapy and future directions in research for the treatment of advanced hormone-refractory prostate cancer. *Cancer Invest* **13**: 296–312

Kroemer G (1997) The proto-oncogene Bcl-2 and its role in regulating apoptosis. *Nature Med* **3(6)**: 614–620

Mackey TJ, Borkowski A, Amin P, Jacobs SC and Kyprianou N (1998) bcl-2/bax ratio as a predictive marker for therapeutic response to radiotherapy in patients with prostate cancer. *Urology* **52(65)**: 1085–1090

McDonnell TJ, Troncoso P, Brisbay SM, Logothetis C, Chung LW, Hsieh JT, Tu SM and Campbell ML (1992) Expression of the proto-oncogene Bcl-2 in the prostate and its association with emergence of androgen-independent prostate cancer. *Cancer Res* **52**: 6940–6944

Mundle SD, Gregory SA, Preislert HD and Raza A (1996) Enzymatic programming of apoptotic cell death. *Pathiobiol* **64**: 161–170

Oltvai ZN, Milliman CL and Korsmeyer SJ (1993) Bcl-2 heterodimerizes *in-vivo* with a conserved homolog, Bax, that accelerates programmed cell death. *Cell* **74**: 609–619

Orth K and Dixit VM (1997) Bik and Bak induce apoptosis downstream of CrmA but upstream of inhibitor of apoptosis. *J Biol Chem* **272**: 8841–8844

Otter I, Conus S, Ravn U, Rager M, Olivier R, Monney L, Fabbro D and Borner C (1998) The binding properties and biological activities of Bcl-2 and Bax in cells exposed to apoptotic stimuli. *J Biol Chem* **273**(11): 6110–6120

Pan G, O'Rourke K and Dixit VM (1998) Caspase-9, Bcl-$X_L$, and Apaf-1 form a terniary complex. *J Biol Chem* **273**(10): 5841–5845

Rokhlin OW, Bishop GA, Hostager BS, Waldschmidt TJ, Sidorenko SP, Pavloff N, Kiefer MC, Umansky SR, Glover RA and Cohen MB (1997) Fas-mediated apoptosis in human prostatic carcinoma cell lines. *Cancer Res* **57**: 1758–1768

Tang DG and Porter AT (1997) Target to apoptosis: a hopeful weapon for prostate cancer. *Prostate* **32**(4): 284–293

Yang E and Korsmeyer SJ (1996) Molecular thanatopsis: a discourse on the Bcl-2 family and cell death. *Blood* **88**: 386–401

Yang E, Zha J, Jockel J, Boise LH, Thompson CB and Korsmeyer SJ (1995) Bad, a heterodimeric partner for *Bcl-$x_L$* and *Bcl-2*, displaces *Bax* and promotes cell death. *Cell* **80**: 285–291

Yin XM, Oltvai ZN and Korsmeyer SJ (1994) BH1 and BH2 domains of Bcl-2 are required for inhibition of apoptosis and heterodimerization with Bax. *Nature* **369**: 321–323

Zha H, Aime-Sempe C, Sato T and Reed JC (1996) Proapoptotic protein Bax heterodimerises with Bcl-2 and homodimerizes with Bax via a novel domain (BH3) distinct from BH1 and BH2. *J Biol Chem* **271**: 7440–7444

Zhou XM, Liu Y, Payne G, Lutz RJ and Chittenden T (2000) Growth factors inactivate the cell death promoter BAD by phosphorylation of its BH3 domain on Ser155. *J Biol Chem* **275**: 25046–25051

Zhu W, Cowie A, Wasfy GW, Penn LZ, Leber B and Andrews DW (1996) Bcl-2 mutants with restricted subcellular location reveal spatially distinct pathways for apoptosis in different cell types. *EMBO J* **15**: 4130–4141

*© 2001 Cancer Research Campaign*

## References

[PDF_00689] Adams J. M., Cory S. (1998). The Bcl-2 protein family: arbiters of cell survival.. Science.

[PDF_00691] Allen R. T., Cluck M. W., Agrawal D. K. (1998). Mechanisms controlling cellular suicide: role of Bcl-2 and caspases.. Cell Mol Life Sci.

[PDF_00694] Berchem G. J., Bosseler M., Sugars L. Y., Voeller H. J., Zeitlin S., Gelmann E. P. (1995). Androgens induce resistance to bcl-2-mediated apoptosis in LNCaP prostate cancer cells.. Cancer Res.

[PDF_00697] Chittenden T., Flemington C., Houghton A. B., Ebb R. G., Gallo G. J., Elangovan B., Chinnadurai G., Lutz R. J. (1995). A conserved domain in Bak, distinct from BH1 and BH2, mediates cell death and protein binding functions.. EMBO J.

[PDF_00701] Colombel M., Symmans F., Gil S., O'Toole K. M., Chopin D., Benson M., Olsson C. A., Korsmeyer S., Buttyan R. (1993). Detection of the apoptosis-suppressing oncoprotein bc1-2 in hormone-refractory human prostate cancers.. Am J Pathol.

[PDF_00705] Diaz J. L., Oltersdorf T., Horne W., McConnell M., Wilson G., Weeks S., Garcia T., Fritz L. C. (1997). A common binding site mediates heterodimerization and homodimerization of Bcl-2 family members.. J Biol Chem.

[PDF_00709] Haldar S., Chintapalli J., Croce C. M. (1996). Taxol induces bcl-2 phosphorylation and death of prostate cancer cells.. Cancer Res.

[PDF_00711] Han J., Sabbatini P., Perez D., Rao L., Modha D., White E. (1996). The E1B 19K protein blocks apoptosis by interacting with and inhibiting the p53-inducible and death-promoting Bax protein.. Genes Dev.

[PDF_00714] Hirotani M., Zhang Y., Fujita N., Naito M., Tsuruo T. (1999). NH2-terminal BH4 domain of Bcl-2 is functional for heterodimerization with Bax and inhibition of apoptosis.. J Biol Chem.

[PDF_00717] Jürgensmeier J. M., Xie Z., Deveraux Q., Ellerby L., Bredesen D., Reed J. C. (1998). Bax directly induces release of cytochrome c from isolated mitochondria.. Proc Natl Acad Sci U S A.

[PDF_00722] Krajewska M., Krajewski S., Epstein J. I., Shabaik A., Sauvageot J., Song K., Kitada S., Reed J. C. (1996). Immunohistochemical analysis of bcl-2, bax, bcl-X, and mcl-1 expression in prostate cancers.. Am J Pathol.

[PDF_00726] Kreis W. (1995). Current chemotherapy and future directions in research for the treatment of advanced hormone-refractory prostate cancer.. Cancer Invest.

[PDF_00729] Kroemer G. (1997). The proto-oncogene Bcl-2 and its role in regulating apoptosis.. Nat Med.

[PDF_00731] Mackey T. J., Borkowski A., Amin P., Jacobs S. C., Kyprianou N. (1998). bcl-2/bax ratio as a predictive marker for therapeutic response to radiotherapy in patients with prostate cancer.. Urology.

[PDF_00734] McDonnell T. J., Troncoso P., Brisbay S. M., Logothetis C., Chung L. W., Hsieh J. T., Tu S. M., Campbell M. L. (1992). Expression of the protooncogene bcl-2 in the prostate and its association with emergence of androgen-independent prostate cancer.. Cancer Res.

[PDF_00738] Mundle S. D., Gregory S. A., Preisler H. D., Raza A. (1996). Enzymatic programming of apoptotic cell death.. Pathobiology.

[PDF_00740] Oltvai Z. N., Milliman C. L., Korsmeyer S. J. (1993). Bcl-2 heterodimerizes in vivo with a conserved homolog, Bax, that accelerates programmed cell death.. Cell.

[PDF_00743] Orth K., Dixit V. M. (1997). Bik and Bak induce apoptosis downstream of CrmA but upstream of inhibitor of apoptosis.. J Biol Chem.

[PDF_00745] Otter I., Conus S., Ravn U., Rager M., Olivier R., Monney L., Fabbro D., Borner C. (1998). The binding properties and biological activities of Bcl-2 and Bax in cells exposed to apoptotic stimuli.. J Biol Chem.

[PDF_00748] Pan G., O'Rourke K., Dixit V. M. (1998). Caspase-9, Bcl-XL, and Apaf-1 form a ternary complex.. J Biol Chem.

[PDF_00751] Rokhlin O. W., Bishop G. A., Hostager B. S., Waldschmidt T. J., Sidorenko S. P., Pavloff N., Kiefer M. C., Umansky S. R., Glover R. A., Cohen M. B. (1997). Fas-mediated apoptosis in human prostatic carcinoma cell lines.. Cancer Res.

[PDF_00754] Tang D. G., Porter A. T. (1997). Target to apoptosis: a hopeful weapon for prostate cancer.. Prostate.

[PDF_00756] Yang E., Korsmeyer S. J. (1996). Molecular thanatopsis: a discourse on the BCL2 family and cell death.. Blood.

[PDF_00758] Yang E., Zha J., Jockel J., Boise L. H., Thompson C. B., Korsmeyer S. J. (1995). Bad, a heterodimeric partner for Bcl-XL and Bcl-2, displaces Bax and promotes cell death.. Cell.

[PDF_00762] Yin X. M., Oltvai Z. N., Korsmeyer S. J. (1994). BH1 and BH2 domains of Bcl-2 are required for inhibition of apoptosis and heterodimerization with Bax.. Nature.

[PDF_00765] Zha H., Aimé-Sempé C., Sato T., Reed J. C. (1996). Proapoptotic protein Bax heterodimerizes with Bcl-2 and homodimerizes with Bax via a novel domain (BH3) distinct from BH1 and BH2.. J Biol Chem.

[PDF_00769] Zhou X. M., Liu Y., Payne G., Lutz R. J., Chittenden T. (2000). Growth factors inactivate the cell death promoter BAD by phosphorylation of its BH3 domain on Ser155.. J Biol Chem.

[PDF_00772] Zhu W., Cowie A., Wasfy G. W., Penn L. Z., Leber B., Andrews D. W. (1996). Bcl-2 mutants with restricted subcellular location reveal spatially distinct pathways for apoptosis in different cell types.. EMBO J.

